# Protein model refinement for cryo-EM maps using *AlphaFold*2 and the DAQ score

**DOI:** 10.1107/S2059798322011676

**Published:** 2023-01-01

**Authors:** Genki Terashi, Xiao Wang, Daisuke Kihara

**Affiliations:** a Department of Biological Sciences, Purdue University, West Lafayette, IN 47907, USA; b Department of Computer Science, Purdue University, West Lafayette, IN 47907, USA; Osaka University, Japan

**Keywords:** cryo-electron microscopy, protein model refinement, protein model validatation, computational methods, *AlphaFold*2, DAQ score

## Abstract

As more protein structure models have been determined from cryogenic electron microscopy (cryo-EM) density maps, establishing how to evaluate the model accuracy and how to correct models in cases where they contain errors is becoming crucial to ensure the quality of the structural models deposited in the public database, the PDB. Here, a new protocol is presented for evaluating a protein model built from a cryo-EM map and applying local structure refinement in the case where the model has potential errors. Firstly, model evaluation is performed using a deep-learning-based model–local map assessment score, DAQ, that has recently been developed. The subsequent local refinement is performed by a modified *AlphaFold*2 procedure, in which a trimmed template model and a trimmed multiple sequence alignment are provided as input to control which structure regions to refine while leaving other more confident regions of the model intact. A benchmark study showed that this protocol, *DAQ-refine*, consistently improves low-quality regions of the initial models. Among 18 refined models generated for an initial structure, DAQ shows a high correlation with model quality and can identify the best accurate model for most of the tested cases. The improvements obtained by *DAQ-refine* were on average larger than other existing methods.

## Abstract 2

A new protocol, *DAQ-refine*, for evaluating a protein model built from a cryo-EM map and applying local structure refinement is described.

## 1. Introduction

Cryogenic electron microscopy (cryo-EM) has become a core technique for determining the three-dimensional structure of biomolecules. Technical advancements in cryo-EM occurred a few years ago which made it possible to achieve near-atomic resolution in determining density maps (Yip *et al.*, 2020 ▸; Nakane *et al.*, 2020 ▸). Cryo-EM has unique strengths including the ability to determine macromolecular structures, such as large complexes or membrane proteins, that are often difficult to crystallize for X-ray crystallography (Cheng, 2018 ▸). As an increasing number of protein structures are being determined by cryo-EM, however, it has been observed that a substantial number of structural models determined using cryo-EM and deposited in the PDB (Berman *et al.*, 2000 ▸) contain potential errors (Terashi *et al.*, 2022 ▸). Errors include cases in which the modeled local conformation itself is wrong and situations in which misassignment of amino acids has occurred in an otherwise correct main-chain conformation.

Structure modeling is intrinsically difficult for map regions in which the local resolution is low. Also, errors often occur when assigning sequences along a long helix, as amino acids in the sequence region all have a high helix propensity. As the ‘resolution revolution’ of cryo-EM (Kühlbrandt, 2014 ▸) has opened the door of structural analysis to many inexperienced users who are attempting to build atomic models into maps of moderate resolution, many mistakes may have been introduced into structural models. Therefore, a reliable protocol is needed that can correctly identify and fix regions in models that are built from a cryo-EM map.

The cryo-EM modeling community recognizes the importance of model validation: it was a focus of discussions in the EM Modeling Challenge held in 2019 (Lawson *et al.*, 2021 ▸). Various model-validation methods have been proposed in the past. Validation methods can be categorized into two main types: map–model scores and model–coordinate scores. The former, map–model scores, measure the consistency of the map and a protein model, and include the atom-inclusion score (Joseph *et al.*, 2017 ▸), *EMRinger* (Barad *et al.*, 2015 ▸), Q-score (Pintilie *et al.*, 2020 ▸) and map correlation score (Joseph *et al.*, 2017 ▸, 2022 ▸). The other category, model–coordinate scores, such as *MolProbity* (Chen *et al.*, 2010 ▸) and *CaBLAM* (Prisant *et al.*, 2020 ▸), identify stereochemical outliers in a protein model. In the worldwide Protein Data Bank (wwPDB), *MolProbity* is used as a validation method for PDB entries. Additionally, the atom-inclusion score and Q-score are used to assess the quality of fit between the map and the atomic structure for cryo-EM data in the PDB. Recently, we developed a novel map–model score, the Deep-learning-based Amino acid-wise model Quality (DAQ) score, which uses deep learning to capture local density features to assess the likelihood that each residue in a model is correct. The DAQ score was very effective in finding the positions in the structure model where amino-acid assignments to local density are likely to be incorrect (residues in the model which were placed more than 2.0 Å away from the native structure; see Supplementary Fig. 3 of Terashi *et al.*, 2022 ▸).

Following our development of the DAQ score, here we address the next step: how to refine local regions that are identified to be incorrect in a structural model. The local refinement protocol, *DAQ-refine*, starts by identifying potentially incorrect regions in a protein model using the DAQ score. It then remodels the local regions using *AlphaFold*2 (AF2), a protein structure-prediction method (Jumper *et al.*, 2021*a* ▸,*b* ▸) that achieved significantly high accuracy in the 14th Critical Assessment of Structure Prediction (CASP14), a community-wide protein structure-prediction experiment (Kryshtafovych *et al.*, 2021 ▸). Although AF2 produces a highly accurate model from the protein sequence information in many cases, it has several reported limitations (Aderinwale *et al.*, 2022 ▸; Jones & Thornton, 2022 ▸). Among the known limitations, the most relevant issue for this work is that a predicted structure from AF2 is built solely based on the sequence and is occasionally different from the conformation in a particular experimental structure and condition, such as in a complex determined by cryo-EM (Zhou *et al.*, 2022 ▸; Heo & Feig, 2022 ▸; Del Alamo *et al.*, 2022 ▸). Thus, in the current refinement protocol, instead of running AF2 as it is, we attempt to keep confident regions in the initial protein model intact and to only remodel low-confidence regions using AF2. This is achieved by following a recently proposed protocol for AF2 in which a partial structure of the target protein is provided as a template to AF2 (Heo & Feig, 2022 ▸).

The current protocol is different and complementary to the protein structure-modeling protocol proposed by Terwilliger and coworkers in the framework of the *Phenix* modeling package (Terwilliger *et al.*, 2022 ▸). Their protocol, *dock_and_rebuild*, employs AF2 and *Phenix* model fitting iteratively to model the entire protein structure model for a density map. In contrast to their approach, the protocol that we discuss here involves correcting local regions in a structural model that has already been built by some modeling tool.

We applied *DAQ-refine* to 13 PDB-deposited models and the corresponding cryo-EM maps reconstructed at 3.20–4.35 Å resolution. Around the 3–5 Å resolution range, structure modeling is intrinsically difficult in map regions where the local resolution is low or moderate. Therefore, it is challenging for model-refinement algorithms. For 11 targets (85%), our local refinement protocol achieved the highest GDT-HA model-assessment score (Kopp *et al.*, 2007 ▸) among four other model-refinement methods. Detailed analysis shows that the final model selection by DAQ(AA) score and the use of a partial model of the target protein as a customized template for AF2 contributed to the high performance of the proposed method. The result demonstrated that *DAQ-refine* is able to identify misaligned regions in the initial models and to refine those regions in an automated fashion.

## 2. Materials and methods

**Figure 1:**
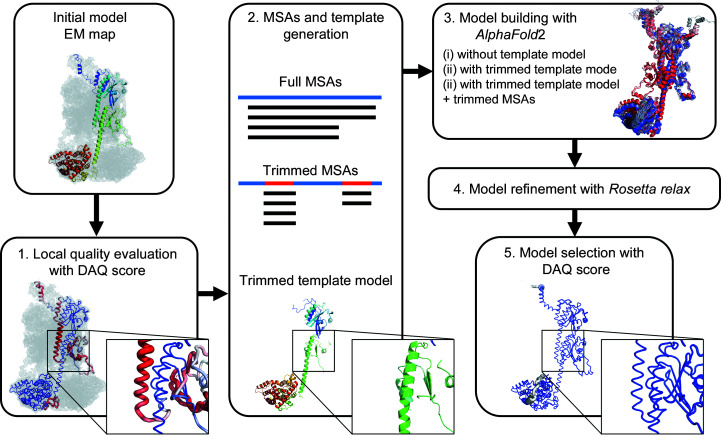
Overview of the *DAQ-refine* protocol. (1) Initial model evaluation with the DAQ score. The initially deposited model for PDB entry 7jsn chain *A*, which was derived from the EM map (EMDB entry EMD-22458, 3.2 Å resolution), is used as an example. The DAQ(AA) scores along the model are shown with a color scale from red [DAQ(AA) < −1.0] to blue [DAQ(AA) > 1.0]. The enlargement highlights regions (residue His230–Glu256) where DAQ(AA) is negative and thus highly likely to be incorrect. (2) MSA and template-model generation. Full MSAs are computed by *MMseqs*2 in *ColabFold*. Trimmed MSAs are generated by masking alignment data corresponding to positions in the full MSAs where the DAQ(AA) score is positive. The trimmed template model is generated by removing residue positions where the DAQ(AA) score is negative or zero from the initial model. (3) Model building by *AlphaFold*2. Three strategies (AF2 with full MSAs, AF2 with full MSAs + trimmed template model and AF2 with trimmed MSAs + trimmed template model) are performed. (4) Models are refined with *Rosetta relax* in the EM map. (5) Finally, the top-ranked model by DAQ(AA) score is selected as the final model.

We illustrate the overall protocol of *DAQ-refine* in Fig. 1 ▸. It consists of five steps.

(i) Initial model evaluation using the DAQ score.
(ii) Generating multiple sequence alignments (MSAs) and a template model as input for AF2. An MSA is a set of aligned similar sequences to the target protein.
(iii) Model building with AF2 with the customized input data. The AF2 neural network directly predicts the 3D co­ordinates of the protein structure using features extracted from the given MSAs and the template model.
(iv) Model refinement with *Rosetta relax*.
(v) Model selection with the DAQ score.

We describe each step in more detail in the subsequent subsections.

### 2.1. Initial model-quality evaluation using the DAQ score

The first step in the protocol is to evaluate the model using the DAQ score. The DAQ score quantifies how well residues in an atomic model agree with density features detected by deep learning. The local density features of an EM map are captured by deep learning. The input EM map is scanned with a box 11 × 11 × 11 Å in size with an interval of 1 Å along the three coordinate-axis directions and a trained deep neural network outputs probabilities that the box contains 20 amino-acid types at the center position of the box (Terashi *et al.*, 2022 ▸).

The DAQ(AA) of a residue position *i* in a protein model is defined as

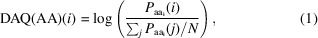

where 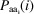 is the computed probability of amino-acid type aa*_i_* being assigned at position *i* by deep learning, which is normalized by the average value among all atom positions *j* in the model. *N* is the total number of atoms in the model. As the equation shows, if the predicted probability value for position *i* is higher than the average, then DAQ(AA) becomes positive. If DAQ(AA) is below −0.5 for an amino-acid position, it is highly likely that the amino acid does not fit the local density and is worth attention. After DAQ(AA) had been computed for all amino acids in a model, the values were averaged by a sliding window of 19 residues along the protein sequence to better detect a local sequence shift in a model (Terashi *et al.*, 2022 ▸).

There are two other types of DAQ score: the probabilities of a C^α^ atom, DAQ(Cα), and of three types of secondary structures, DAQ(SS), being in a scanning box. Here, we only use DAQ(AA) since it detects sequence-assignment shifts and incorrect conformations in a structure model, which are two common errors in protein structure modeling.

### 2.2. Running *AlphaFold*2 with modified MSAs and template

Low-confidence regions detected by the DAQ score in the initial model were refined with AF2. In this local refinement, we want to only refine the local regions and keep confident regions in the model untouched. A regular AF2 run is not suitable for this task because it will generate a full residue model from the sequence information only, which may deviate in the confident regions of the initial model and consequently may not fit well to the map. To achieve local refinement with AF2, we modified the input to AF2 in two different ways and compared the results with a regular AF2 run. Thus, we performed three different runs. We used *ColabFold* (Mirdita *et al.*, 2022 ▸) on Google Colab to run AF2 because the server allows us to use customized input.

A regular AF2 run takes an input protein sequence, generates an MSA with *MMseqs*2 (Mirdita *et al.*, 2019 ▸; Steinegger & Söding, 2017 ▸), which we call here a full MSA, and runs AF2. AF2 simply performs structure prediction and does not consider the initial structural model. This procedure produced five models.

The second run uses the full MSA with a trimmed initial model as a template, in which residues with a negative DAQ(AA) score, and potentially incorrectly modeled residue positions, are removed from the initial model. The intention of the trimmed template model is to provide a template that only covers confident regions of the initial model. In AF2 trained network models, only two fine-tuned models (model 1 and model 2) use template data (Jumper *et al.*, 2021*a* ▸). Therefore, this run produces two models from model 1 and model 2.

The third run is with a trimmed MSA and the trimmed template model. In the trimmed MSA, we mask local regions in the full MSA that correspond to residues with a zero or positive DAQ(AA) score, *i.e.* confident regions in the initial model (AF2 with trimmed MSA + trimmed template model). The intention of the trimmed MSA is to not provide MSA information for the confident regions of the model, so that AF2 does not have information to alter the structure of the confident regions. This procedure also produces two models. In total, we have nine models for a target protein from the three different AF2 runs. The strategy to trim an MSA and a template was inspired by work by Heo and Feig in which they attempted to produce multiple conformations of target proteins by controlling the MSA and template to input (Heo & Feig, 2022 ▸).

The nine models were superimposed on the trimmed template model, which was then subjected to the *Rosetta relax* protocol (Nivón *et al.*, 2013 ▸; Conway *et al.*, 2014 ▸) with the cryo-EM density map. In the *Rosetta relax* protocol, the agreement of the atomic mode and experimental density on the 3D map is used as an additional energy term for structure refinement. Finally, 18 models, nine models with and nine without *Rosetta* relaxation, were evaluated using the DAQ(AA) score. We selected the model that has the highest DAQ(AA) score as the final model.

### 2.3. Data set of PDB entries to refine

We tested the *DAQ-refine* protocol on 13 PDB chain models with corresponding EM maps, which were taken from the data sets used in the DAQ score paper (Terashi *et al.*, 2022 ▸). These targets were selected because their initial model has mismodelled regions as indicated by negative DAQ(AA) scores.

Six of the 13 targets (PDB entries 6cp3 chain *Y*, 6k1h chain *Z*, 7jsn chain *A*, 7jsn chain *B*, 7ksm chain *C* and 7ksm chain *D*) were PDB entries that have at least two versions of the deposited structure available in the wwPDB database (http://ftp-versioned.wwpdb.org). Moreover, the two versions of the structures differ by a C^α^ r.m.s.d. of greater than 1.0 Å and the first version has a mismodelled region that has a low negative DAQ(AA) score. The second version of the structure has a substantially better DAQ(AA) score with a positive value, indicating that the error in the model was fixed by the authors. We rebuilt the model from the initial version of the structure and compared it with the second version of the deposited structure. These six targets were named the 2Ver targets. The targets and file names of the PDB files on the PDB FTP site are listed in Supplementary Table S1.

The other seven targets (PDB entries 6l54 chain *C*, 3j6b chain 9, 5lc5 chain *N*, 6gcs chain 2, 6gcs chain 4, 6cv9 chain *A* and 6iqw chain *E*) were selected from protein structure pairs from different PDB entries (and corresponding different EMDB entries) that have over 90% sequence identity to each other but nevertheless have a C^α^ r.m.s.d. of 1.0 Å or higher and have at least four contiguous inconsistent and thus probably misaligned residues between them. We defined these misaligned residues to be corresponding residues in the two structures that are more than 2.0 Å away from each other and are close to different residues when the two models were superimposed. We selected pairs in which one member of the pairs had a negative DAQ(AA) score for that region. For a structure pair, we refined the structure with lower DAQ score using the associated EM map and examined whether the refined structure became close to the higher-scoring counterpart. We call these targets homologous pair (Hom) targets.

There is a possibility that both entries of a Hom target are correct since they are not identical proteins and have been independently deposited in the PDB. However, one of the pair has a negative DAQ(AA) score, which usually indicates an incorrect model, and our local refinement protocol constantly improved DAQ(AA) when applied to the entry with the lower score. Thus, as we also discussed in the original DAQ paper (Terashi *et al.*, 2022 ▸), we believe that the entry with the lower score does indeed include incorrectly modeled regions. The EMDB and PDB IDs of these pairs of targets are provided in Supplementary Table S2. In Supplementary Table S3, we provide the average DAQ(AA) scores of each of two structures of the 13 targets as well as the r.m.s.d.s between them.

## 3. Results and discussion

### 3.1. Local refinement results with the three *AlphaFold*2 protocols

We first show a summary of the refined structures built by the three AF2 protocols discussed above: AF2 with a full MSA and no template, AF2 with a full MSA and a trimmed template model, and AF2 with a trimmed MSA and a trimmed template model. After each model had been generated, we ran the *Rosetta relax* protocol. For comparison, we also show the results of applying *Rosetta relax* only to the initial models. The results are presented as two metrics: the root-mean-square deviation of C^α^ atoms (r.m.s.d.) and the high-accuracy version of the global distance test (GDT-HA; Kopp *et al.*, 2007 ▸) to the native (*i.e.* the second version model for the 2Ver targets and the higher scored structure for the Hom targets). GDT-HA is defined as the average fraction of correctly modeled C^α^-atom positions obtained in each superposition to the native structure at four distance thresholds: 0.5, 1.0, 2.0 and 4.0 Å. Table 1 ▸ shows the r.m.s.d. and GDT-HA of the best model among those generated for each protocol. Supplementary Tables S4 and S5 provide the results for all 18 models for each target.

When *Rosetta relax* was directly applied to the initial models, improvement was observed for six targets and no change was observed for one target (53.8% considering both cases) out of the 13 targets when r.m.s.d. was considered. GDT-HA improved for four cases, with no change for four other cases out of 13. The average GDT-HA decreased slightly from 0.69 to 0.68. For both the improvements in r.m.s.d. and GDT-HA, the Wilcoxon test indicates high *p*-values of 0.63 and 0.58, respectively. Thus, using only *Rosetta relax* did not give a statistically significant improvement. This is mainly because misaligned residues in the initial models are difficult to resolve by regular structure refinement.

When we applied the three AF2 protocols followed by *Rosetta relax*, the resulting models clearly improved over the simple application of structure relaxation (Table 1 ▸). The average r.m.s.d. improved from 3.33 to 1.42 and 1.33 Å for AF2 with a full MSA and no template model and for AF2 with a full MSA and a trimmed template model, respectively. The average r.m.s.d. became worse for AF2 with a trimmed MSA and a trimmed template model, due to two targets, PDB entry 6gcs chain 2 and PDB entry 6cv9 chain *A*, where the resulting r.m.s.d. increased. The r.m.s.d. became very large for these two targets because the refined structures had a tail region that was placed on the wrong side of the protein structure (Supplementary Fig. S1). In terms of GDT-HA, all three *DAQ-refine* protocols improved the initial models except for one target, PDB entry 6k1h chain *Z*. Comparing the three *DAQ-refine* protocols, AF2 with a full MSA and a trimmed template model performed the best, with the smallest average r.m.s.d. and the largest average GDT-HA.

**Figure 2:**
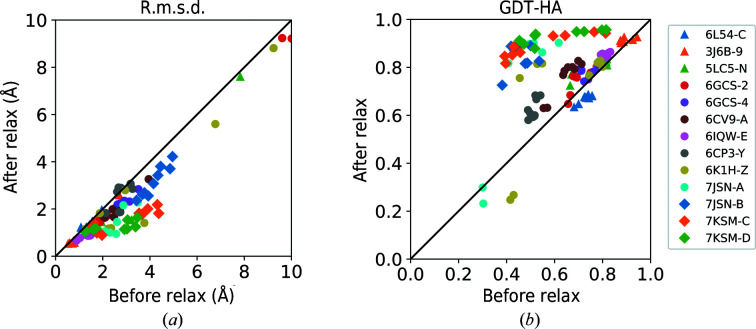
Model quality before and after refinement. R.m.s.d. and GDT-HA for nine models of all 13 targets before and after applying *Rosetta relax* structure refinement are shown. (*a*) R.m.s.d.; (*b*), GDT-HA. For r.m.s.d., four models were omitted from the plot that have r.m.s.d. values of over 10 Å. They were two models from PDB entry 6cv9 chain *A*, which have before and after r.m.s.d. values of 26.0 and 26.2 Å and of 35.7 and 35.5 Å, respectively, and two models from PDB entry 7jsn chain *A*, which have before and after r.m.s.d. values of 26.0 and 25.6 Å and of 25.4 and 25.1 Å, respectively.

To further examine the effect of *Rosetta relax*, in Fig. 2 ▸ we compare the model accuracy before and after applying *Rosetta relax*. The relaxation improved the models in most of the cases. 110 of 117 models (94.0%) improved and one model stayed the same in terms of r.m.s.d., while 100 models (85.5%) improved in terms of GDT-HA using the structure-relaxation step. For both r.m.s.d. and GDT-HA, the Wilcoxon test result indicates that the improvements are statistically significant (*p*-values of 1.7 × 10^−19^ and 2.2 × 10^−14^, respectively). Thus, *Rosetta relax* is not helpful when used on its own, but is effective in combination with AF2 runs, *i.e.* the full *DAQ-refine* protocol.

### 3.2. Model selection with the DAQ score

**Figure 3:**
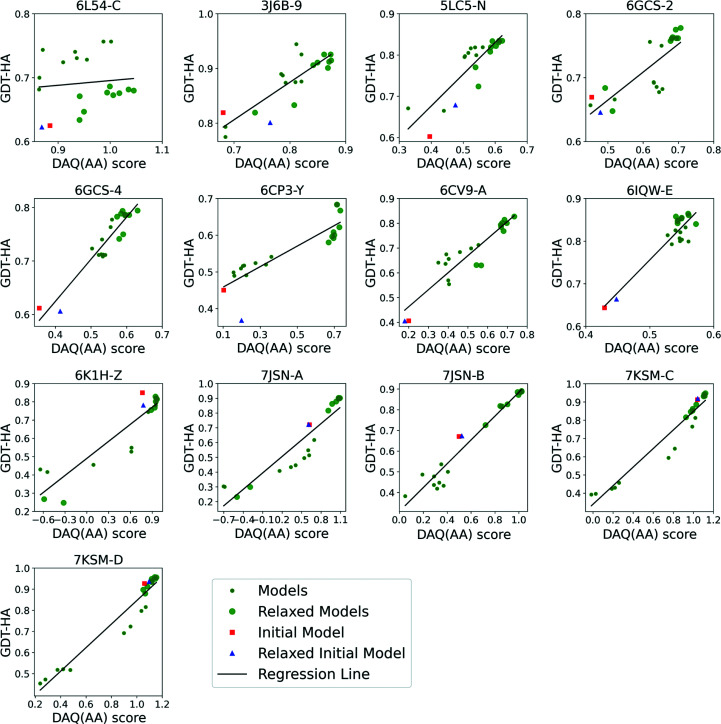
Comparison of the DAQ(AA) score and GDT-HA for 13 targets. The DAQ(AA) scores of 18 structure models refined with our protocol are plotted against GDT-HA. Small green circles represent models refined without the *Rosetta relax* protocol. Large green circles represent the refined models after the *Rosetta relax* protocol. Initial models and initial models relaxed using *Rosetta relax* are shown as red squares and blue triangles, respectively. Black lines represent the regression lines of the plots.

The next question that we address is how to select the refined structures from the 18 models that we have built. In Fig. 3 ▸ we show GDT-HA for each of the 18 generated models, including unrelaxed and relaxed models, for each target relative to the DAQ(AA) score. For reference, the initial model and the relaxed initial model from *Rosetta relax* are also included in the plots. The same plots instead showing r.m.s.d. are provided as Supplementary Fig. S2. The plots show that DAQ(AA) has a clear correlation with the model quality GDT-HA. For each target, the Pearson correlation coefficient and a regression line between GDT-HA and DAQ(AA) were computed from 20 models (18 generated models, the initial model and the relaxed initial model). The Pearson correlation coefficients ranged from 0.10 (PDB entry 6l54 chain *C*) to 0.97 (PDB entries 7jsn chain *B*, 7ksm chain *C* and 7ksm chain *D*), with an average of 0.85. Except for PDB entry 6l54 chain *C*, all targets have a correlation coefficient of over 0.85. Therefore, we can use DAQ(AA) as a metric to select one of the most accurate models from those generated.

For PDB entry 6l54 chain *C*, *Rosetta relax* made GDT-HA worse in all generated models (Figs. 2 ▸ *b* and 3 ▸). Close examination of the EM map of the target structure (EMDB entry EMD-0837) and the map of the homologous protein structure which was considered as the reference (PDB entry 6z3r chain *C*; EMDB entry EMD-11063) revealed that the two EM maps differ slightly in the local region where PDB entry 6l54 chain *C* contains a modeling error (Supplementary Fig. S3). Therefore, *Rosetta relax* further deviated the structures away from PDB entry 6z3r chain *C* towards the density of EMDB entry EMD-0837, which made GDT-HA lower; moreover, DAQ(AA) was better for the refined models with lower GDT-HA because they agree better with the density of EMDB entry EMD-0837.

**Figure 4:**
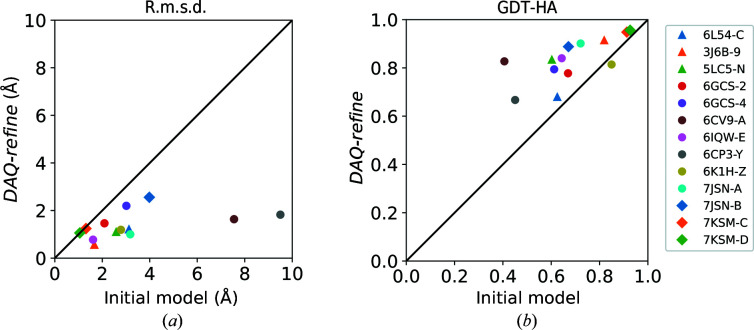
Model selection with DAQ(AA). From 18 models generated for each target, that with the highest DAQ(AA) was selected. The selected models were compared with the initial model in terms of (*a*) r.m.s.d. and (*b*) GDT-HA.

Fig. 4 ▸ shows actual model-selection results using DAQ(AA). The model with the highest DAQ(AA) was improved over the initial model in terms of both r.m.s.d. and GDT-HA (Figs. 4 ▸ *a* and 4 ▸ *b*). There was one target, PDB entry 7ksm chain *D*, for which the model with the highest DAQ(AA) has the same r.m.s.d. as the initial model. In terms of GDT-HA, a model worse than the initial model was selected for another target, PDB entry 6kih chain *Z*. The average improvement in r.m.s.d. was 1.96 Å, while the improvement in GDT-HA from the initial models was on average 0.15. The Wilcoxon test indicates statistical significance for the improvements in both r.m.s.d. and GDT-HA, with low *p*-values of 4.9 × 10^−4^ and 1.2 × 10^−3^, respectively.

### 3.3. Comparison with other structure-refinement methods

We further compared the *DAQ-refine* protocol with four other existing refinement methods, namely molecular-dynamics flexible fitting (MDFF) with a *g*-scale of 0.5 (Singharoy *et al.*, 2016 ▸; McGreevy *et al.*, 2016 ▸), the *Rosetta relax* protocol (Nivón *et al.*, 2013 ▸; Conway *et al.*, 2014 ▸), *phenix.real_space_refine* (Afonine *et al.*, 2013 ▸) and *phenix.dock_and_rebuild* (Terwilliger *et al.*, 2022 ▸). MDFF performs structure refinement with molecular dynamics under the constraint of an input density map. *phenix.real_space_refine* performs gradient-driven minimization of the target function that combines the fit of the model to the map and the restraints on the protein structure, such as bond lengths and angles. *phenix.dock_and_rebuild* first runs AF2 to predict the tertiary structure of the target protein and then splits it into reliable domains based on the pLDDT score; it then performs docking and rebuilding iteratively for each domain in the map. Additionally, a simple combination of AF2 and *Rosetta relax* was also implemented for comparison. This protocol used a full MSA and the initial structural model as input to AF2. The generated model with the best pLDDT score was then superimposed on the initial structural model and refined with *Rosetta relax*.

**Figure 5:**
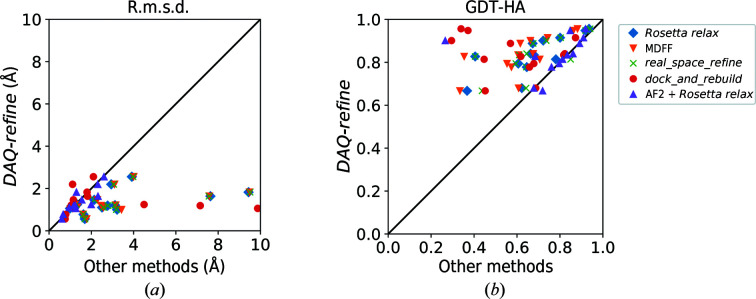
Comparison of *DAQ-refine* with five other existing methods. For each of the 13 targets the model with the highest score was selected from the models generated by each method. Blue diamonds, *Rosetta relax*; orange triangles, MDFF; green crosses, *phenix.real_space_refine*; red circles, *phenix.dock_and_rebuild*; purple triangles, AF2 + *Rosetta relax*. The *phenix.dock_and_rebuild* and AF2 + *Rosetta relax* protocols start by predicting the structure of the target protein using AF2. AF2 + *Rosetta relax* uses the initial protein model as a template protein structure in AF2. The other refinement methods started from the initial protein model. For our *DAQ-refine* protocol, the model with the highest DAQ(AA) score was selected. (*a*) Comparison is made in terms of r.m.s.d. to the native structure. Two models generated by *phenix.dock_and_rebuild* and AF2 + *Rosetta relax* have large r.m.s.d.s. of 25.6 and 24.8 Å and were not included in this plot. (*b*) Comparison is made in terms of GDT-HA.

Fig. 5 ▸ compares the r.m.s.d. and GDT-HA of refined structures for the 13 targets from the *DAQ-refine* protocol and the results from the five existing methods. Numerical values of the data are provided in Supplementary Table S6. ‘DAQ-refine’ in Fig. 5 ▸ and ‘Top Score Model’ in Supplementary Table S6 are the final model that has the highest DAQ(AA) score among the 18 generated models from the *DAQ-refine* protocol. It should be noted that *dock_and_rebuild* only constructs models for confident regions, as shown in Supplementary Table S7. On average, models from *dock_and_rebuild* cover 89% of the amino acids in the target proteins; the rest were not modeled. Therefore, models generated by *dock_and_rebuild* tend to have low r.m.s.d.s but a low coverage that results in a low GDT-HA.

Our protocol achieved a lower r.m.s.d. for all of the targets when compared with *Rosetta relax*, MDFF and *phenix.real_space_refine* (Fig. 5 ▸ *a*). When compared with models from *phenix.dock_and_rebuild*, *DAQ-refine* had a larger r.m.s.d. than *phenix.dock_and_rebuild* for six target models (PDB entries 6l54 chain *C*, 5lc5 chain *N*, 6gcs chain 2, 6gcs chain 4, 6cp3 chain *Y* and 7jsn chain *B*), but this is mainly because *phenix.dock_and_rebuild* did not model all of the residues in the proteins, *i.e.* its models are shorter than the native structure. Compared with AF2 + *Rosetta relax*, our protocol achieved a lower r.m.s.d. for nine targets (69.2%). When GDT-HA is considered, *DAQ-refine* showed a higher value for all of the targets than *Rosetta relax* and MDFF. Compared with *phenix.real_space_refine* and *phenix.dock_and_rebuild*, our protocol showed a higher GDT-HA for 12 targets (92.3%). Compared with AF2 + *Rosetta relax*, *DAQ-refine* showed a higher or same GDT-HA for 11 targets.

### 3.4. Case studies

The following sections discuss four case studies that illustrate how *DAQ-refin*e improved the initial models.

#### 3.4.1. Case study 1: PDB entry 7jsn chain *A* (EMDB entry EMD-22458)

**Figure 6:**
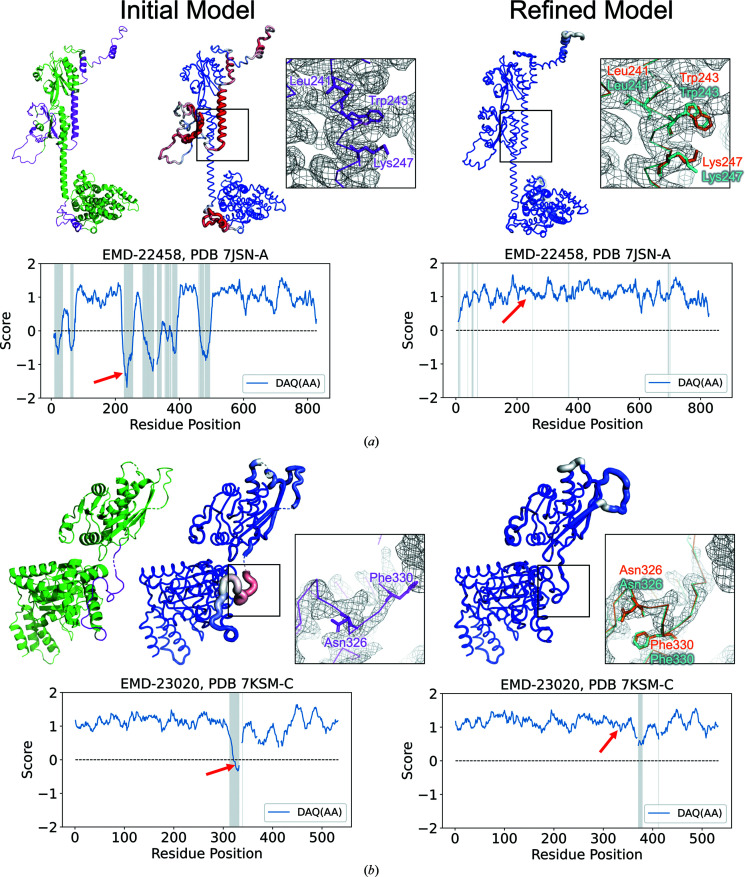
Analysis of initial and refined models of two 2Ver targets using the DAQ(AA) score. Left: the initial model (the first version of the PDB entry). The color of the chain reflects the deviation of C^α^-atom positions from the native structure (the revised version of the entry). Color is scaled from green (deviation < 1.0 Å) to magenta (deviation > 3.0 Å). Middle and right: the initial and refined models colored by the DAQ(AA) score. The color is scaled from red [DAQ(AA) < −1.0] to blue [DAQ(AA) > 1.0]. The radius of the chain tube is thicker if the region has a low DAQ(AA) score. The enlargements show model regions with a low DAQ(AA) score. The initial, refined and revised models are indicated in magenta, cyan and orange, respectively. Surface meshes represent the EM map at the author’s recommended contour level. Plots show the DAQ(AA) scores along the sequence position. Gray in the plot represents residue positions where the deviation of the C^α^-atom position between the model and native (the revised structure) is larger than 3.0 Å. (*a*) PDB entry 7jsn chain *A*, which was built from the EM map (EMDB entry EMD-22458). A region containing Leu241, Trp243 and Ly247, which has a residue shift in sequence assignment in the initial model, is enlarged. The corresponding position in the plot is indicated by a red arrow. In the refined model, residue conformations in the native (the revised model) are shown in orange and cyan residues are those refined by *DAQ-refine*. (*b*) PDB entry 7ksm chain *C* with the EM map (EMDB entry EMD-23020) from which the chain structure was built. A region with a residue shift in the initial model, which includes Asn326 and Phe330, is enlarged.

In this example, we refined the first version of the model of cGMP-specific 3′,5′-cyclic GMP phosphodi­esterase (cGMP phosphodiesterase 6 subunit; PDB entry 7jsn chain *B*; Gao *et al.*, 2020 ▸; Fig. 6 ▸ *a*). The model was built from a 3.2 Å resolution EM map (EMDB entry EMD-22458). PDB entry 7jsn contains two versions of the structure. The first model was released in the PDB on 21 October 2020, and was later revised on 31 March 2021 (Gao *et al.*, 2021 ▸). In the initial model, which is shown in the left panel of Fig. 6 ▸(*a*), there are several regions colored magenta where large deviations of over 3.0 Å were observed when compared with the revised version of the model. The DAQ(AA) score clearly indicates low values for these inconsistent regions, as shown in red in the second panel from the left.

The refined structure is shown on the right in Fig. 6 ▸(*a*). The DAQ(AA) score of the refined model is much improved, with positive values for all of the residues, as indicated in blue in the model. The change in the scores along the residue positions is clear in the two plots shown below the model structure images. In the initial model, inconsistent regions with the native (shown in gray) had low, negative DAQ(AA) scores, while the refined model has all positive DAQ(AA) values along the chain. One of the remodeled regions is highlighted in squares. In the initial model, residues including Leu241, Trp243 and Lys247 do not fit well to the density. The problem with this region is that the assigned sequence was shifted along the helix. On the other hand, in the refined model shown on the right, the revised conformation of the three residues (cyan) agree well with the native structure except for the direction of the tip of Lys247. After local refinement, the overall model accuracy was improved from 3.18 to 1.00 Å in C^α^ r.m.s.d. and from 0.72 to 0.90 in GDT-HA.

#### 3.4.2. Case study 2: PDB entry 7ksm chain *C* (EMDB entry EMD-23020)

The second example is a model of the human mitochondrial AAA+ protein LONP1. The first version of the model (PDB entry 7ksm; Shin *et al.*, 2021 ▸) was released in the PDB on 2 December 2020 and it was revised on 15 June 2022. As shown in the left plots in Fig. 6 ▸(*b*), there is an inconsistent region (Ile312–Glu339, gray shade in the score plot and shown in magenta in the leftmost model) between the first and revised versions of the model, where DAQ(AA) exhibited negative values as indicated by a thick tube in red. As highlighted in the square, the side chains of Phe330 and Asn326 in the first version of the model (magenta) were not covered well by the map density. The main problem in this region is that the sequence assignment was shifted along the main-chain conformation, which resulted in the unnatural side-chain conformation in the density. In the refined model (right panel) DAQ(AA) for these two amino-acid residues improved to positive values and they fit well into the map. After refinement, the overall model accuracy improved from 1.31 to 1.24 Å in C^α^ r.m.s.d. and from 0.91 to 0.95 in GDT-HA.

#### 3.4.3. Case study 3: PDB entry 6l54 chain *C* (EMDB entry EMD-0837)

**Figure 7:**
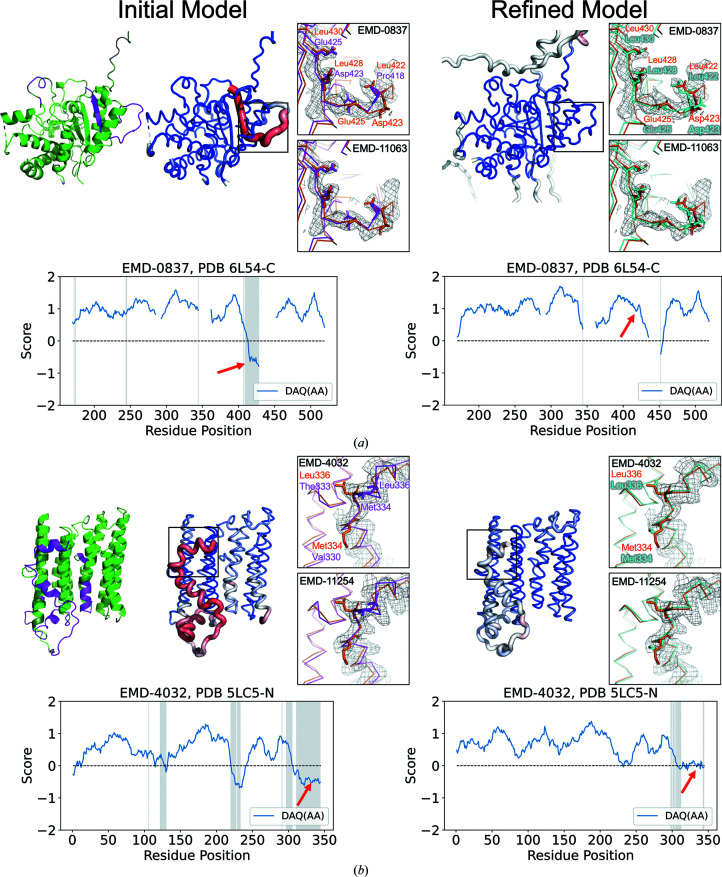
Analysis of initial and refined models of two Hom targets using the DAQ(AA) score. (*a*) PDB entry 6l54 chain *C* built from EMDB entry EMD-0837 contained regions that are inconsistent with the other protein model, PDB entry 6z3r chain *C* from EMDB entry EMD-11063. This region is shown in magenta in the leftmost structure and is detected as negative DAQ(AA) scores, as shown in red with thick tubes in the model second from the left. The refined model is shown on the right. It has an overall positive score (blue). The four squares provide magnified views of residues in the inconsistent region before and after refinement by *DAQ-refine*. EMDB entry EMD-0837 used for refinement is shown as a mesh in the top squares. The lower squares show the map of the reference homologous structure (EMDB entry EMD-11063). The DAQ score plots have gaps at missing residues in the initial model (PDB entry 6l54 chain *C*). In the enlargements, Asp423 and Glu425 in pink are the conformations of these two residues in the initial model, whereas those in cyan are the results of *DAQ-refine*. Those in orange are conformations in the reference structure, PDB entry 6z3r chain *C*. (*b*) Local refinement of PDB entry 5lc5 chain *N*, which was built from EMDB entry EMD-4032, in comparison with PDB entry 6zkm chain *N*, which was derived from EMDB entry EMD-11254. The enlargements highlight Met334 and Leu336 in an inconsistent region between the two entries. The squares show the maps of the initial structure and the reference homologous structure (top, EMD-4032; bottom, EMD-11254) as meshes. In the refined model, Met334 and Leu336 were shifted to the corresponding positions in PDB entry 6zkm chain *N* and the DAQ(AA) score improved (red arrows in the plots).

The next two examples are taken from the Hom targets. Fig. 7 ▸(*a*) is a pair of models of the protein SMG9. One is PDB entry 6l54 chain *C*, which was modeled from EMDB entry EMD-0837, determined at 3.43 Å resolution (Zhu *et al.*, 2019 ▸) and the other is PDB entry 6z3r chain *C*, built from EMDB entry EMD-11063, at 2.97 Å resolution (Langer *et al.*, 2020 ▸). These two protein models have 100% identical sequences, yet their structures deviate by an r.m.s.d. of 3.1 Å. In PDB entry 6l54 chain *C*, Thr405–Leu428 have negative DAQ(AA) scores (shown in red in the second model from the left), indicating a likely shift in the sequence assignment. On close inspection, it is observed that this region has implausible side-chain packing. For example, the side chains of Asp423 and Glu425 are directed into the hydrophobic core of the protein in PDB entry 6l54 (magenta, highlighted in the enlargement). However, in PDB entry 6z3r these residues are exposed to solvent (orange model, highlighted in the square on the right), which would be more appropriate. PDB entry 6l54 chain *C* has a total DAQ(AA) score of 272.4, which is lower than the DAQ(AA) score of 308.2 for PDB entry 6z3r chain *C*. Therefore, we used PDB entry 6l54 chain *C* as the initial model and refined it to see whether the structure becomes closer to PDB entry 6z3r chain *C* as the reference structure. As shown in the right column in Fig. 7 ▸(*a*), *DAQ-refine* corrected the inconsistent region and improved the DAQ(AA) score. After local refinement, Asp423 and Glu425 in PDB entry 6l54 chain *C* (cyan in the enlargement on the right) adopted similar conformations to those in PDB entry 6z3r chain *C* (orange). Reflecting this change, the DAQ(AA) score improved to positive values, as shown in the plots. The map–model cross-correlations also improved from 0.442 to 0.528 for the initial and refined structures, respectively. Supplementary Figure S4(*a*) shows sequence and structure alignments of the initial and refined models. In the initial model, two phenylalanine residues, Phe414 and Phe421, are not covered by the density of the map and fit better into the density in the refined model. The EM map does not have density after Leu428 of the initial model, which would be a possible reason why the large shift of about five residues occurred and was possible in the initial model.

#### 3.4.4. Case study 4: PDB entry 5lc5 chain *N* (EMDB entry EMD-4032)

The last example (Fig. 7 ▸ *b*) is a pair of structures of NADH-ubiquinone oxidoreductase chain 2: PDB entry 5lc5 chain *N* (EMDB entry EMD-4032, 4.35 Å resolution; Zhu *et al.*, 2016 ▸) and PDB entry 6zkm chain *N* (EMDB entry EMD-11254, 2.8 Å resolution; Kampjut & Sazanov, 2020 ▸). These two protein models have a high sequence identity of 91.9% but have an r.m.s.d. of 2.6 Å. There are three inconsistent regions between the two PDB models, which are indicated in magenta in the leftmost structure model and in gray shades in the DAQ(AA) plot on the left. These regions are identified as having a low, negative DAQ(AA) score. The DAQ(AA) score clearly indicates a potential misalignment at Lys311–Val344 in PDB entry 5lc5 chain *N*. Applying the refinement protocol to PDB entry 5lc5 chain *N* modified the sequence assignment of this region and improved the DAQ(AA) from negative values to positive, as shown in the two plots (the positions indicated by red arrows). The refined model of PDB entry 5lc5 chain *N* has now an identical sequence assignment to PDB entry 6zkm chain *N*. To illustrate the refined model, we show two residues: Leu336 and Met334. In the initial model, PDB entry 5lc5 chain *N*, these two hydrophobic residues are facing solvent and do not fit well into the density. The refined structure (cyan in the enlargement on the right) now has these two residues in the same conformations as in PDB entry 6zkm chain *N*, where they face the interior of the protein and make hydrophobic interactions, which would be more reasonable. In this region the authors only positioned C^β^ atoms, probably because the side-chain densities were not clearly visible within the author-recommended contour level. This low resolution probably prevented detection of the misalignment in the initial structure by the authors. Supplementary Figure S4(*b*) highlights the region of the initial and refined models in the map (EMDB entry EMD-4032).

In all four of these examples the DAQ(AA) score detects inconsistent regions between two compared models by a negative score in one of the models, and these regions were improved to a positive DAQ(AA) score by the *DAQ-refine* protocol.

## 4. Summary

As more protein structure models built from high-resolution EM maps become available, accurate model evaluation and suitable local refinement methods become more important. In this work, we present *DAQ-refine*, a protein structure local refinement protocol which uses local model-quality evaluation with the DAQ score to detect potential locally incorrect regions and then rebuilds them with *AlphaFold*2. To reflect the local data quality from the DAQ score in the refinement step by *AlphaFold*2, we introduced a trimmed template model and trimmed MSAs. Trimmed input data allow *AlphaFold*2 to focus on building incorrect regions while keeping correct regions almost intact. Comparing the three *DAQ-refine* protocols, AF2 with a full MSA and a trimmed template model performed the best, with the smallest average r.m.s.d. and the largest average GDT-HA. Our protocol generates a series of different models using different *AlphaFold*2 settings. We demonstrated that the DAQ(AA) score has a substantial correlation with the quality of the models and is able to select good models among those generated.

## 5. Availability

The *DAQ* program is freely available for academic use via GitHub (https://github.com/kiharalab/DAQ). In addition, the *DAQ* program is available to run on a Google Colab notebook at https://bit.ly/daq-score and https://github.com/kiharalab/DAQ/blob/main/DAQ_Score.ipynb. *DAQ-refine* including the modified *ColabFold* that can use both trimmed MSAs and a trimmed template model is available at https://bit.ly/DAQ-Refine and https://github.com/kiharalab/DAQ-Refine. This Colab notebook provides the instructions for *DAQ-refine* and tools for generating trimmed template models and trimmed MSAs based on the results of the *DAQ* program.

## Supplementary Material

Supplementary Tables S1 and S2 and Supplementary Figures. DOI: 10.1107/S2059798322011676/ji5028sup1.pdf

Supplementary Tables S3, S4, S5, S6 and S7. DOI: 10.1107/S2059798322011676/ji5028sup2.xlsx

## Figures and Tables

**Table 1:** Summary of structure remodeling with the three *AlphaFold*2-based methods The EMDB IDs for entries in the Hom data set are indicated with an asterisk (*). The other six entries are from the 2Ver data set. R.m.s.d. and GDT-HA were computed for the whole model including all residues of the protein. The results of three variations of running *AlphaFold*2 after structure refinement with *Rosetta relax* are shown. The ‘Relax only’ column shows the results of applying the *Rosetta relax* protocol to the initial structure. No templ., no template model; trim. templ., trimmed template model; trim. MSA, trimmed MSA. Each method outputs multiple models and the best result among the outputs is listed here. A triangle (▴) is shown when the result is worse than the initial model. The best values for each target are indicated in bold.

		R.m.s.d. (Å)					GDT-HA				
EMD ID	PDB ID	Initial	Relax only	Full MSA + no templ.	Full MSA + trim. templ.	Trim. MSA + trim. templ.	Initial	Relax only	Full MSA + no templ.	Full MSA + trim. templ.	Trim. MSA + trim. templ.
EMD-0837*	6l54 chain *C*	3.12	3.12	1.23	**1.21**	1.73	0.63	0.62▴	**0.69**	0.68	0.65
EMD-2566*	3j6b chain 9	1.67	1.69▴	0.54	**0.53**	1.76▴	0.82	0.80▴	**0.93**	**0.93**	0.83
EMD-4032*	5lc5 chain *N*	2.58	2.51	**1.02**	1.19	1.53	0.60	0.68	**0.83**	0.82	0.77
EMD-4384*	6gcs chain 2	2.09	2.14▴	1.62	**1.46**	9.22▴	0.67	0.65▴	0.76	**0.78**	0.68
EMD-4384*	6gcs chain 4	3.01	2.94	**2.13**	2.20	2.32	0.61	0.61	0.79	**0.79**	0.75
EMD-7637*	6cv9 chain *A*	7.55	7.64▴	1.66	**1.64**	26.2▴	0.41	0.41	0.80	**0.83**	0.63
EMD-9708*	6iqw chain *E*	1.61	1.63▴	0.87	**0.67**	0.75	0.64	0.66	0.86	**0.86**	0.84
EMD-7546	6cp3 chain *Y*	9.50	9.44	**1.83**	1.86	2.85	0.45	0.37▴	**0.68**	**0.68**	0.62
EMD-9906	6k1h chain *Z*	2.79	2.77	1.17	**1.15**	1.82	0.85	0.78▴	0.82▴	**0.83**▴	0.80▴
EMD-22458	7jsn chain *A*	3.18	3.22▴	**0.95**	1.00	1.05	0.72	0.72	0.90	**0.90**	0.90
EMD-22458	7jsn chain *B*	3.98	3.92	2.56	**2.36**	3.80	0.67	0.67	0.89	**0.89**	0.83
EMD-23020	7ksm chain *C*	1.31	1.33▴	1.77▴	**0.89**	1.24	0.91	0.92	0.89	0.93	**0.95**
EMD-23020	7ksm chain* D*	1.05	0.99	1.14▴	1.13▴	**1.04**	0.93	0.94	0.94	0.95	**0.96**
Average		3.34	3.33	1.42	**1.33**	4.25▴	0.69	0.68▴	0.83	**0.84**	0.79
