# Supplementary material for: Protein model refinement for cryo-EM maps using AlphaFold2 and the DAQ score
Source: Acta Crystallogr D Struct Biol. 2023 Jan 1;79(Pt 1):10–21. doi: 10.1107/S2059798322011676 (PMC9815095; doi:10.1107/S2059798322011676)
Supplement: Supplementary file 1 [file d-79-00010-sup1.pdf]

STRUCTURAL BIOLOGY

Volume 79 (2023)

Supporting information for article:

Protein model refinement for cryo-EM maps using *AlphaFold*2 and the DAQ score

Genki Terashi, Xiao Wang and Daisuke Kihara

Supplementary Table S1. Six EMDB entries and protein chains in the 2Ver targets.

EMID	resolution (Å)	PDB ID	CIF file of the first version	CIF file of the revised version
7546	3.8	6CP3-Y	pdb_00006cp3_xyz_v1.cif	pdb_00006cp3_xyz_v2.cif
9906	3.52	6K1H-Z	pdb_00006k1h_xyz_v1.cif	pdb_00006k1h_xyz_v2.cif
22458	3.2	7JSN-A	pdb_00007jsn_xyz_v1.cif	pdb_00007jsn_xyz_v2.cif
22458	3.2	7JSN-B	pdb_00007jsn_xyz_v1.cif	pdb_00007jsn_xyz_v2.cif
23020	3.2	7KSM-C	pdb_00007ksm_xyz_v1.cif	pdb_00007ksm_xyz_v2.cif
23020	3.2	7KSM-D	pdb_00007ksm_xyz_v1.cif	pdb_00007ksm_xyz_v2.cif

Each target has two versions of the protein structure. The fifth and sixth columns show the file names of the deposited models in the wwPDB database (ftp-versioned.wwpdb.org).

Supplementary Table S2. Seven protein chain pairs in Homologous pair targets.

Initial Model			Native Model			
EMID	Resolution (Å)	PDB ID	EMID	Resolution (Å)	PDB-ID	SeqID (%)
0837	3.43	6L54-C	11063	2.97	6Z3R-C	100.0
2566	3.2	3J6B-9	3556	3.2	5MRF-9	99.5
4032	4.35	5LC5-N	11254	2.8	6ZKM-N	91.9
4384	4.32	6GCS-2	4872	3.3	6RFQ-2	100.0
4384	4.32	6GCS-4	4872	3.3	6RFQ-4	100.0
7637	3.8	6CV9-A	11674	3.62	7A6U-A	97.4
9708	3.35	6IQW-E	9255	3.1	6MUT-E	100.0

Each target has two protein chains. For each target, the initial model has a lower DAQ score than the native model and used as the starting model for remodeling. The native model is the structure, which the initial model and remodeled structure were compared against. The average DAQ(AA) score and RMSD between the initial model and native models are shown in Supplementary Table S3. The percentage of the sequence identities between the initial and native model (SeqID(%)) was computed by the align command in Pymol.

Supplementary Table S3. Model quality and DAQ(AA) score of the initial model and the native models of the targets. (in a separate Excel file).

Each target has two structures. The initial model is the model to which the remodeling protocols were applied. The native model is the other model, which was considered as the correct structure of the protein of the initial model.

Supplementary Table S4. RMSD of all remodelled structures by three AF2-based protocols (in a separate Excel file).

For each of the 13 targets, RMSD of the models built by the three AF2-based protocols are shown. RMSD of before and after Rosetta Relax refinement are shown.

Supplementary Table S5. GDT-HA of all remodelled structures by three AF2-based protocols (in a separate Excel file).

For each of the 13 targets, GDT-HA of the models built by the three AF2-based protocols are shown. GDT-HA of before and after Rosetta Relax refinement are shown.

Supplementary Table S6. RMSD and GDT-HA of models built by four existing remodelling protocols (in a separate Excel file).

Numerical values of remodelled structures of the 13 targets by the four methods, Rosetta Relax, MDFF, phenix.real_space_refine, phenix.dock_and_build, and the combined protocol of AF2 and Rosetta Relax are shown. These data were used to plot Fig. 5.

Supplementary Table S7. Coverage of models generated by phenix.dock_and_build (in a separate Excel file).

A model built by phenix.dock_and_build does not necessarily include all the residues in the target because the procedure starts from fitting reliable regions of a AF2 model into the density map. In this table, the fraction of modelled residues in each target is shown.

Supplementary Figure S1. Two examples of the AF2 with a trimmed MSA and a trimmed template model

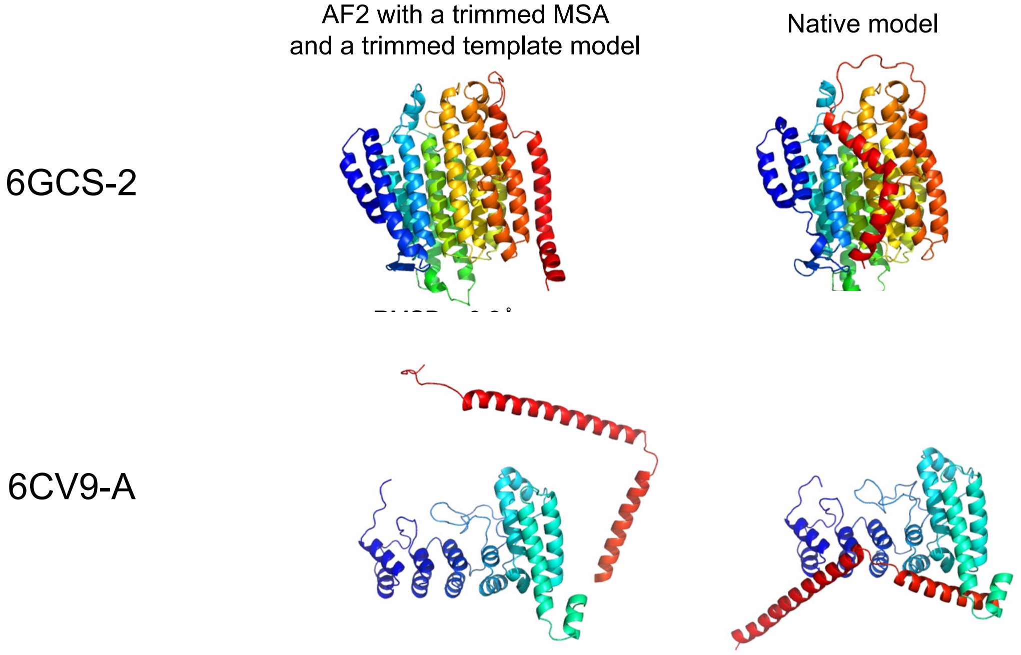

Models are colored from blue to red from the N- to C-terminal residues. The RMSD between the refined and native models became very large for 6GCS-2 and 6CV9-A. Compared with native models (right column), the refined structures (left column) had a C-terminal tail region (red) that was placed on the wrong side of the protein structure.

Supplementary Figure S2. Comparisons of DAQ(AA) score and Ca-RMSD of models for the 13 targets.

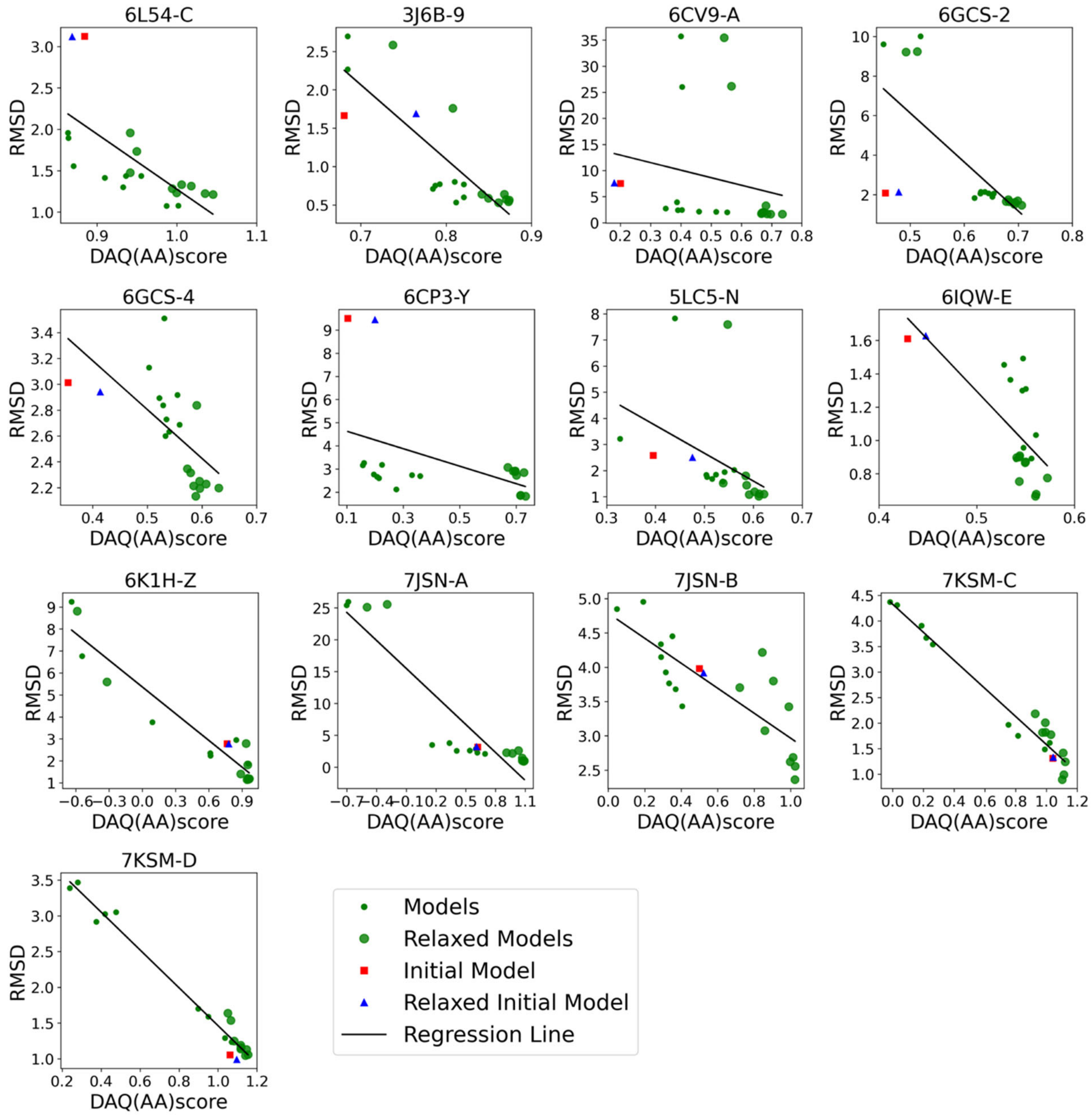

Small green circles represent the refined models without the Rosetta relaxation protocol. Large green circles represent the refined models after the Rosetta relaxation protocol. Initial models and relaxed initial models

by Rosetta relaxation are shown by red square and blue triangle, respectively. Black lines represent the regression line of the plots.

Supplementary Figure S3. Analysis of EM maps for the target 6L54-C

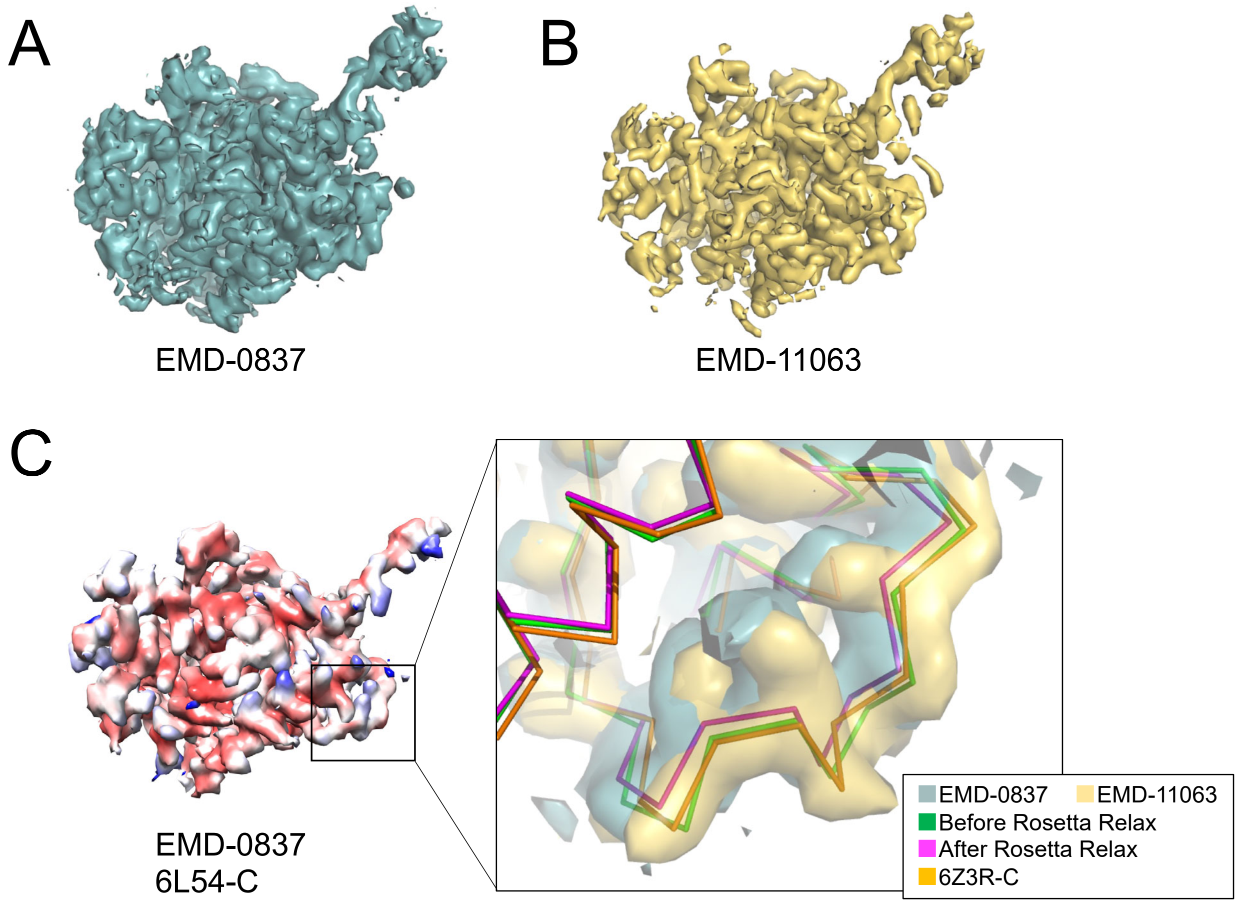

Here we closely examined two EM maps for the target 6L54-C. **A.** The EM map of EMD-0837, which is associated with 6L54-C. The author-recommended contour level is used for visualization. **B.** The EM map, EMD-11063, which is associated with the homologous protein, 6Z3R-C (reference structure). **C.** Colors on the map surface represent the map-map local correlation between the two maps. The local correlation was computed by UCSF Chimera's "*vop local Correlation*" command. Colors are scaled from blue (local correlation < 0.0) to red (local correlation = 1.0). EMD-0837 shows negative local correlations (blue) with EMD-11063 at outer loop regions, where the 6L54-C has modeling error. In the magnified image, the orange tube model represents the reference structure (PDB 6Z3R-C). Green and magenta tube models represent models before and after applying Rosetta Relax, respectively. The blue and yellow maps are EMD-0837 and EMD-11063, respectively. The Rosetta Relax changed the loop structure (Cys411-Ser424) toward the map of the initial structure, EMD-0837, further away from 6Z3R-C. Consequently, GDT-HA dropped from 0.76 to 0.68 since 6Z3R-C is considered as the reference.

Supplementary Figure S4. Analysis of initial refined models of two Hom targets.

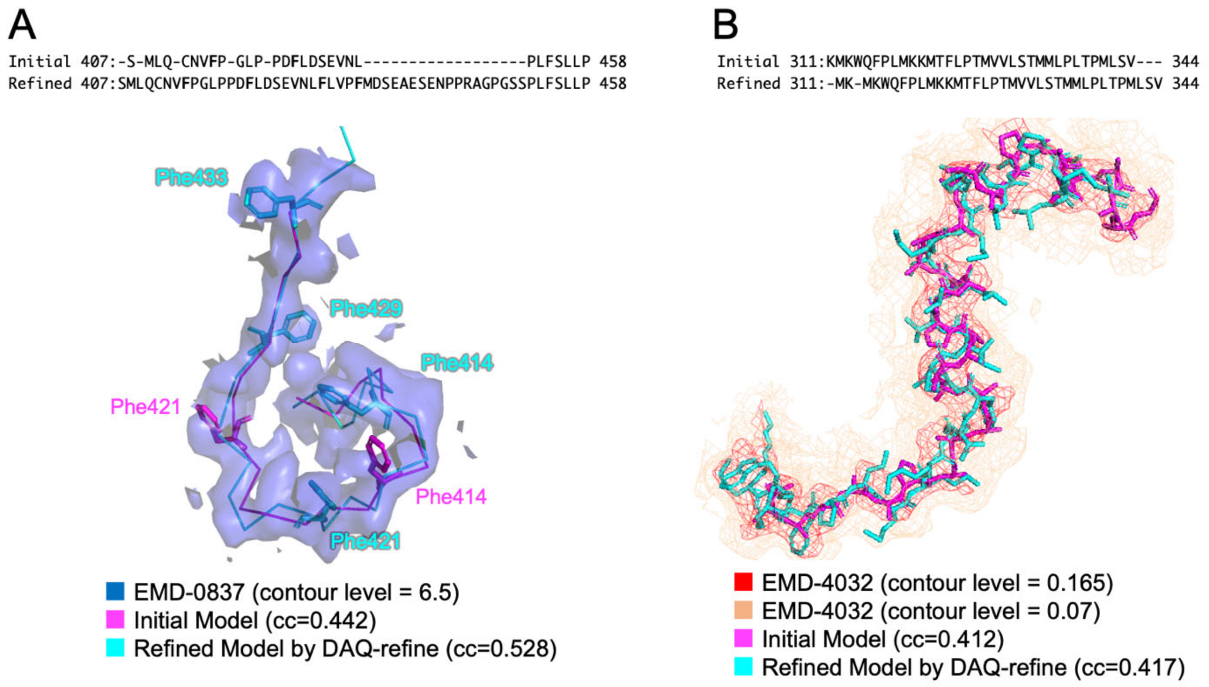

The top alignments show the structural alignment between the initial and refined models.

A. EMD-0837. Corresponds to Figure 7A**. B.** EMD-4032. Corresponds to Figure 7B.
